# Dissecting Calcific Aortic Valve Disease—The Role, Etiology, and Drivers of Valvular Fibrosis

**DOI:** 10.3389/fcvm.2021.660797

**Published:** 2021-05-10

**Authors:** Petra Büttner, Lukas Feistner, Philipp Lurz, Holger Thiele, Joshua D. Hutcheson, Florian Schlotter

**Affiliations:** ^1^Department of Internal Medicine/Cardiology, Heart Center Leipzig at University of Leipzig, Leipzig, Germany; ^2^Department of Biomedical Engineering, Florida International University, Miami, FL, United States; ^3^Biomolecular Sciences Institute, Florida International University, Miami, FL, United States

**Keywords:** aortic stenosis, CAVD, fibrosis, pathogenesis, myofibroblast, sex differences

## Abstract

Calcific aortic valve disease (CAVD) is a highly prevalent and progressive disorder that ultimately causes gradual narrowing of the left ventricular outflow orifice with ensuing devastating hemodynamic effects on the heart. Calcific mineral accumulation is the hallmark pathology defining this process; however, fibrotic extracellular matrix (ECM) remodeling that leads to extensive deposition of fibrous connective tissue and distortion of the valvular microarchitecture similarly has major biomechanical and functional consequences for heart valve function. Significant advances have been made to unravel the complex mechanisms that govern these active, cell-mediated processes, yet the interplay between fibrosis and calcification and the individual contribution to progressive extracellular matrix stiffening require further clarification. Specifically, we discuss (1) the valvular biomechanics and layered ECM composition, (2) patterns in the cellular contribution, temporal onset, and risk factors for valvular fibrosis, (3) imaging valvular fibrosis, (4) biomechanical implications of valvular fibrosis, and (5) molecular mechanisms promoting fibrotic tissue remodeling and the possibility of reverse remodeling. This review explores our current understanding of the cellular and molecular drivers of fibrogenesis and the pathophysiological role of fibrosis in CAVD.

## Introduction

Aortic valve stenosis (AS) is a devastating disorder characterized by progressive narrowing of the aortic valve (AV) orifice and has a high prevalence (1) exceeding 2% in a population ≥75 years of age (2). AS is the leading cause for interventional or surgical heart valve therapy (3). AV sclerosis, defined by leaflet thickening through extracellular matrix (ECM) expansion, serves as a precursor for AS (4, 5). The rate of progression from AV sclerosis to AS is elevated (6, 7); however, once any degree of stenosis is present, progression to severe stenosis is common (8). Even early-stage AV sclerosis associates with increased cardiovascular morbidity and mortality (9, 10), and recent clinical studies have suggested that intervention prior to late-stage stenosis may improve overall cardiovascular outcomes (11–13). Myofibroblast activation, collagen accumulation, proteoglycan degradation, and elastic fiber fragmentation represent the hallmark processes underlying fibrotic valve remodeling leading to AV sclerosis. In addition, lipid and lipoprotein accumulation, inflammatory cell infiltration, and tissue calcification are contributing pathomechanisms (5, 14–17). While tissue fibrosis plays a major role in the initiation phase of calcific aortic valve disease (CAVD), the differential contribution of fibrosis and calcification to late-stage AS is less clear. This conundrum is exemplified by the larger relative contribution of fibrosis to the same hemodynamically defined degree of AS in women compared with men (18, 19). Uncovering mechanisms promoting AV fibrosis has the potential to lead to the development of therapeutic interventions targeting the early stage of CAVD and may address the need for sex-specific treatment paths.

## AV Biomechanics and ECM Composition

Heart valve movement is passive, following blood pressure changes in the cardiac chambers. The valvular structure is uniquely designed to bend with its curvature, while restricting bending against it (20). Physiological blood flow across the heart valves is unidirectional. For the AV, the valve opens when left ventricular (LV) pressure exceeds aortic pressure during systole and closes when LV pressure falls below the aortic pressure during diastole. The primary role of the AV is to prevent backflow of blood into the LV during diastole, as this would lead to ventricular volume overload. During diastole, a back pressure of ~80 mmHg acts on the 1-mm thick AV (21, 22). The high valvular collagen content is the primary structural element bearing this tensile force and shifts the load to the aortic wall (21). When subjected to this pressure gradient, the leaflet's strain is anisotropic (i.e., different in circumferential and radial directions) and reaches ~25% in the radial direction and ~10% in the circumferential direction (23).

**Figure 1:**
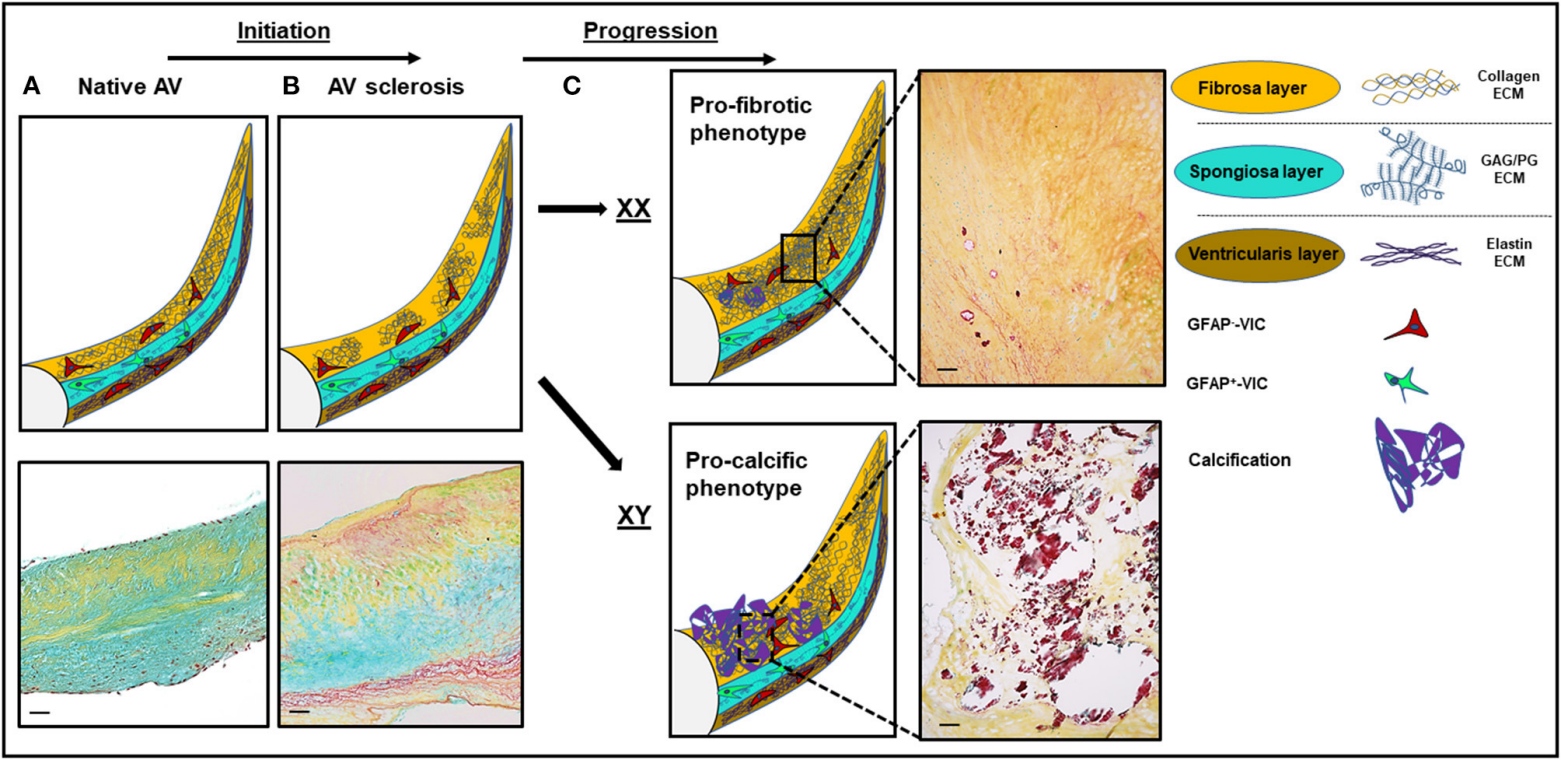
Structural and histological changes in calcific aortic valve disease (CAVD). **(A)** The native aortic valve (AV) has three distinct extracellular matrix (ECM) layers: the fibrosa (yellow), the spongiosa (turquoise), and the ventricularis (dark yellow). VICs in the spongiosa layer express GFAP (48). **(B)** Disease initiation in CAVD is marked by fibrotic ECM expansion and disarray in the fibrosa layer. **(C)** Disease progression is sex-dependent: a more profibrotic phenotype in women and a more procalcific phenotype in men. Movat staining: yellow, collagen; turquoise, proteoglycans; dark purple, calcification. Scale bars indicate 100 μm. GFAP, glial fibrillary acidic protein. GAG, glycoaminoglycans; PG, proteoglycans; XX, female; XY, male.

The AV's ECM architecture is a fine-tuned, specialized structure designed to withstand these extreme biomechanical stresses across the span of a lifetime. During that time span, all valves open and close ~3 × 10^9^ times, highlighting the valve's intrinsic ability to withstand mechanical fatigue by continual ECM remodeling (24, 25). The AV is composed of three layers perfectly adjusted to meet varying biomechanical needs: a dense layer of stress-bearing, circumferentially oriented collagen extending into the valvular supporting structures toward the aortic outflow side (fibrosa layer, Figure 1A); a second elastin-rich layer with sparce radially aligned collagen adjacent to the inflow surface responsible for diastolic recoil and rapid closure of the valve (ventricularis layer); and a third layer, the spongiosa, situated in-between the other two layers having sponge-like, shear-absorbing properties owing to its high glycosamino-(GAG)- and proteoglycan content. It mediates and absorbs the dynamic mechanical forces that the valve microstructure endures during the cardiac cycle and provides resistance against compressive forces (25).

Collagens are the major constituent of the AV's ECM, comprising 50% of dry weight (26) and are markedly increased in fibrosis. Elastin is the second most prevalent protein comprising 13% of the AVs dry weight (26). Collagen type I (74%), type II (24%), and type V (2%) are the most abundant collagens present in the AV (25). Collagen fibers can be stretched but cannot be compressed, whereas elastin fibers can stretch and contract (21). Consequently, it seems obvious that alterations in ECM composition affect valve biomechanics, but the contribution of individual ECM components to AS remains incompletely understood (27).

During systole, the ventricular side is passed by the laminar blood flow jet as it exits the valve at a velocity of ~1 m/s creating shear stress peaking at 60–80 dyn/cm^2^ (28). In contrary, on the fibrosa side, turbulent, oscillatory flow creates a low shear environment peaking at diastole (15–20 dyn/cm^2^) (22, 29). However, one study suggested mostly unidirectional, non-oscillatory shear stress on the aortic side of the valve due to sinus vortices (29). These vortices may not develop when diastolic coronary blood flow is present (30). Shear stresses are lowest on the fibrosa side of the non-coronary leaflet due to the lack of diastolic coronary flow (4), and interestingly, the non-coronary leaflet has the highest susceptibility to calcification (4).

## Patterns in the Cellular Contribution, Temporal Onset, and Risk Factors for Valvular Fibrosis

### Cellular Heterogeneity

The AV layer architecture is dramatically dependent on specialized cells that generate and maintain the ECM components. The AV is populated by two major cell types: valvular endothelial cells (VECs) that line the aortic and ventricular border of the AV and valvular interstitial cells (VICs) that regulate the ECM and maintain homeostasis (31, 32). VICs are positive for the mesenchymal lineage marker vimentin (VIM) (31, 33). VECs carry the markers PECAM1 (CD31) and VWF (34).

Three major VIC phenotypes exist: (i) quiescent VICs that associate with low ECM remodeling, (ii) activated myofibroblastic VICs that trigger profound ECM remodeling, and (iii) osteoblastic VICs that contribute to ECM mineralization (35). In normal heart valves, the majority of VICs are quiescent (~90%), and the rate of myofibroblastic VICs is <10% (31, 36). Myofibrogenesis is a hallmark process in valvular heart disease (37), and the proportion of myofibroblastic VICs dramatically increases under pathological conditions (33).

Myofibroblastic VIC activation is characterized by the expression of α-smooth muscle actin (α-SMA) and its organization in contractile stress fibers (31, 32, 38). α-SMA enables these cells to contract and migrate. Myofibroblastic VICs are further characterized by their central role in ECM remodeling, as they synthesize ECM components and remodeling enzymes [matrix metalloproteinases (MMPs), most predominantly MMP-2], tissue inhibitors of metalloproteinases (TIMPs), and cathepsins (35, 39). Under physiological conditions, myofibroblast activation is terminated by apoptosis and de-differentiation to a quiescent state (37). Osteoblastic VICs appear to generate from a myofibroblast state (38).

Novel evidence suggests that beyond these classical valve cell populations, a broad array of additional specialized cells contribute to the heterogeneous cellular environment of the AV. In a single-cell RNA sequencing (scRNA-seq) analysis, cells were defined as VICs if COL1A1 and COL3A1 expression was present (40). The majority of cells, however, were categorized as valve-derived stromal cells (VDSCs), as they failed to match previously identified VEC and VIC markers (40). Of note, the prototypical mesenchymal lineage and VIC marker vimentin (VIM) (31, 33) was not considered in this analysis, potentially underscoring the rate of heterogeneity within the VIC population, as collagen expression may primarily represent myofibroblastic activation (41).

VECs are essential for valvular homeostasis and structural integrity (42), shielding the valvular ECM and VICs from cells (e.g. immune cells) and signaling molecules in the blood stream and from hemodynamic forces. By means of endothelial-to-mesenchymal transdifferentiation (EndMT), VECs contribute to the VIC population especially during embryogenesis (43). However, this process still plays a role in valve homeostasis and disease during postnatal and adult life (42, 44, 45). As the resident VIC population can prevent VEC EndMT (42), it was suggested that VEC subpopulations could act as a reservoir for VIC replenishment (44) when the reduction in vital VICs indicates it (46). Moreover, VECs were shown to undergo osteogenic differentiation promoting calcium deposition *in vitro* (42). Osteogenic differentiation potential was also shown for mitral VECs (47). Of note, EndMT leading to a myofibroblastic phenotype appears to precede differentiation of VECs to an osteoblastic phenotype (42), suggesting that VECs may actively contribute to sclerosis and calcification of the valve in addition to their protective role as a barrier against the invasion of pathological mediators into the valvular interstitium.

The high level of cellular heterogeneity is also signified by the identification of cellular markers that reflect the layered architecture of the AV. As such, glial fibrillary acidic protein (GFAP) was identified as a unique marker of VICs from the spongiosa layer (48). Furthermore, in fibrotic AV tissues, enrichment of GFAP RNA and protein expression were noted (48). These findings point toward an active role of these spongiosa VICs in AV remodeling and fibrosis generation. This is underpinned by the observation that the largest increase in collagen fiber number in CAVD/AS occurs in the spongiosa layer (49). The highest abundance of α-SMA protein expression representative for myofibroblastic VICs was noted in the ventricularis AV layer (48) that matches with side-dependent mechanobiological responses in VICs as previously shown (50). Side-dependent differences in gene expression profiles were also observed in VECs (51, 52). Interestingly, the side dependency extends to the cellular orientation of VECs, which were shown to align with the underlying collagen architecture (53).

### Temporal Onset (Initiation and Progression of Sclerosis and Fibrosis)

Early on, the focus in CAVD research shifted from the perception of AS as an unmodifiable condition to underlying modifiable active disease processes (5). Aortic valve thickening (sclerosis) represents an initial feature of the CAVD spectrum (Figure 1B). The prevalence of AV sclerosis follows a linear relationship with age (10). In study populations <60 years of age, AV sclerosis is infrequent (<10%) (10). However, the proportion increases, exceeding 50% in those 80 years and older (10). Histopathologically, these early sclerotic lesions, primarily located in the base region on the aortic side of the leaflet, are characterized by lipid and inflammatory cell infiltration and ECM expansion (4, 5). ECM remodeling is facilitated by proteolytic enzymes (54) from macrophages and myofibroblasts as well as synthesis of ECM components (55, 56).

Maintenance of collagen homeostasis is crucial for valve function (27). In fibrotic wound healing, alterations in synthesis vs. catabolism of the ECM regulate the net increase or decrease in collagen (57). When synthesis of collagen by myofibroblasts exceeds the rate at which it is degraded, the total amount of collagen increases over time and fibrosis occurs (58). Collagen synthesis is elevated in myofibroblastic VICs (41). In the native valve, net collagen content increases with age (59), but the collagen protein fraction decreases with age (60), as other non-collagenous ECM proteins like proteoglycans (biglycan, decorin, osteoglycin) and the glycoprotein fibronectin 1 increase in abundance in parallel (48). Collagen *de novo* synthesis is counteracted by collagen-degrading proteolytic enzymes. Among these, metalloproteinases and cathepsins are the major proteolytic enzyme classes (33, 61–63). Ensuing collagen fragmentation and degradation compromises the AV's structural integrity in the same way that exceeding synthesis does. GAGs are involved in CAVD (64–69); however, their degree of contribution to fibrosis remains unclear. Progression to advanced and hemodynamically severe aortic stenosis is hallmarked by the emergence of large calcific nodules and a net loss of collagen relative to the total ECM protein (41). It is important to note that the role of fibrosis in altering aortic valve biomechanics during disease progression remains unclear. Previous studies have suggested that fibrosis precedes calcification in CAVD and stiffens the leaflets at early stages. As noted, fibrosis may serve as the predominant pathological feature in women with AS. Future studies are needed to delineate the relative contribution of fibrosis and calcific minerals in changing aortic valve biomechanics during disease progression. This may require the advent of animal models that better recapitulate the progression of the human disease, as the analysis of the entire CAVD pathological spectrum in human samples is limited by sample accessibility.

### AV Sclerosis Risk Factors, the Profibrotic AS Phenotype in Women, and a Special Role of Fibrosis in Bicuspid Aortic Valve Disease

An array of risk factors specifically for AV sclerosis exist. Older age, male sex, smoking, and arterial hypertension were identified as independent predictors of AV sclerosis in the Cardiovascular Health Study (70). Higher levels of the atherogenic lipoprotein a [lp(a)] also relate to AV sclerosis (71). One study suggested an association of serum total cholesterol levels >200 mg/dl, hypertension, diabetes mellitus, and serum high density cholesterol levels <35 mg/dl with calcified or thickened aortic leaflets or root (72). Higher serum phosphate levels within the normal range were associated with AV sclerosis (73). Light to moderate alcohol consumption was associated with lower odds of AV sclerosis (74). AV sclerosis itself is a risk factor associated with adverse cardiovascular outcomes (9, 10, 75).

In women, severe disease based on fibrotic degeneration is more frequently observed than the calcific phenotype that predominates in men (19), suggesting that rather than the typical paradigm of a profibrotic remodeling progressing to calcification, female valve disease may result from a continuation of collagen accumulation (Figure 1C). Stenotic aortic valves explanted from women show less calcification and denser connective tissue characteristic of fibrosis than valves from men regardless of age and phenotype (bicuspid vs. tricuspid) (18). Importantly, biological sex appears to play a major role in VIC gene expression and biology (76, 77). *In vitro*, male VICs showed a higher alkaline phosphatase content and higher propensity for calcification (76). Sex-specific features and mechanisms in CAVD have been reviewed extensively before (78, 79). Of note, most reports focus on calcification as the primary outcome, and little is known about sex-dependent differential effects on fibrosis. In cell culture experiments using cultivated rat VICs, male VICs produced more collagen I, GAG, MMP2, and alkaline phosphatase and showed higher calcific nodule size and calcified area. However, VIC proliferation rate was significantly higher in female porcine and rat VICs (76). In a microarray-based analysis on porcine VICs, VICs from women were less likely to express molecular signatures related to proliferation, inflammation, and apoptosis (77). These two analyses provide a differing signal toward cellular proliferation (76, 77). While results from *in vivo* and *in vitro* analysis may vary, the observed differences *in vitro* could relate to serum-free cell culture conditions. In a biomarker study in humans, women had a higher propensity for biomarkers representing fibrosis and men for biomarkers signifying inflammation and calcification (80). The PI3K/Akt signaling pathway has been shown to play a sex-specific role in human VICs, leading to increased calcification when inhibited (81). The secretion of the two profibrotic interleukins (ILs) IL-6 and IL-8 was elevated in VICs from men relative to VICs from women after interferon-α and lipopolysaccharide treatment (81). Another study reported a more robust activation of extracellular signal-regulated kinases (ERKs) and hypoxia-inducible factor-alpha (HIF-1α) via signal transducer and activator of transcription-1 (STAT-1) pathways in male human VICs (82). Interestingly, expression levels of the ECM-degrading enzyme MMP-1 were elevated in male VICs (82). The global phenotypic and transcriptional differences likely are the result of a complex mix of intrinsic (chromosomal complement) and extrinsic factors (sex hormones) (78).

Bicuspid aortic valve (BAV) disease is the most prevalent congenital heart defect affecting ~1–2% of the population and frequently leads to early-onset CAVD and ensuing valve degeneration (83, 84). The hemodynamic consequence may either be regurgitation or stenosis (21). Bicuspid and tricuspid CAVD share fibrosis and calcification as disease-defining processes (18). Differences in fibrotic tissue composition and calcification between BAVs and TAVs primarily were evident within male or female sexes (18). Aortic valve calcification density as determined by computed tomography (CT) was significantly higher in older men (i.e. >60 years) with BAV than men with TAV and younger men with BAV (18). No significant differences were observed for the percentage of dense connective tissue and collagen fibers as markers of fibrosis between BAVs and TAVs (18). However, another histological study reported a higher grade of valvular fibrosis in stenotic BAVs vs. TAVs (85). On the molecular level, the ECM-degrading protease MMP-2 was shown to have higher expression levels in BAVs than TAVs (86). Strikingly, in all congenital semilunar valve disease, including BAV, the layered valve microstructure is disrupted (87). The ECM disorganization, increased ECM production, and VIC disarray likely is the result of defective developmental programs resulting in tissue fibrosis (88).

## Imaging Valvular Fibrosis

Given the noted sex-dependent differences and clinical data suggesting improved outcomes with earlier intervention, modalities are needed to diagnose early-stage aortic valve remodeling. Detection of tissue fibrosis in the AV, however, remains a challenge. The early lesion of CAVD, AV sclerosis, can be detected by echocardiography (10, 89) and CT as leaflet thickening, indicating fibrotically remodeled AV tissue. Transthoracic echocardiography is the cornerstone imaging modality to assess valvular morphology and function and to grade AS severity. Temporal resolution is high and the spatial resolution allows for the detection of leaflet thickening and macrocalcification. Due to the superior acoustic window, transesophageal echocardiography may be used to characterize calcification distribution (90). However, assessment of ECM composition and microcalcification is constrained by limited spatial resolution in both modalities. CT is the primary imaging modality to assess calcification burden (90–92), owing to its excellent spatial resolution and high discriminatory power to detect calcification (92) but not other tissue components. Direct evaluation of tissue changes would be the ideal diagnostic tool to monitor disease development and progression and to determine end points for evaluation of therapeutic interventions (93). Molecular probes for the imaging of fibrosis are under clinical investigation (94). However, they have not been evaluated for the assessment of valvular fibrosis. Both inflammation and developing calcification can be detected through positron emission tomography (PET)-CT (95), and PET-CT may be able to detect microcalcifications (96).

## Biomechanical Consequences of Valvular Fibrosis

The valvular microenvironment is defined by a complex interplay of the layer structure, ECM composition, cells, cellular differentiation states, and secretory profile of specific cell types. All these elements are subject to dynamic, high-velocity forces that the AV endures. ECM stiffness is defined by ECM composition, plays a pivotal role in this microenvironment, and defines force transmission from the ECM to the cells and the interaction of cells with the ECM (97). As ECM composition varies across the valve layers, so does tissue stiffness. Tissue stiffness therein defines the extent of resistance to deformation. The onset of tissue fibrosis dramatically alters mechanical properties.

The fibrosa layer is on average two times stiffer than the ventricularis (98, 99) and the spongiosa (100). In addition, focal regions in the fibrosa are stiffer than any part of the ventricularis (98). AV stiffness increases with age and correlates with collagen content (59). Perturbation of this fine-tuned system leads to inhomogeneous stress, and force distribution across the valve leaflet and pathological positive feedback loops develop. In these feedback loops, mechanosensitive cells like VICs undergo myofibroblastic activation and contribute to increased tissue stiffness and thickness (5) through elevated secretion of disorganized collagen (41) and ECM-modifying proteases. Matrix stiffness itself is sufficient to modulate VIC phenotypes without further stimulation (101–103). When cultured on stiff substrates, VICs express more α-SMA-positive stress fibers (36, 101, 103). The mechanosensitivity even extends to the level of individual VIC cellular stiffness (24). VICs from left-sided heart valves, residing in an environment with high transvalvular pressures, have higher cellular stiffness compared to VICs from the right-sided heart valves (24).

Myofibroblastic VICs can contract and thereby alter their biomechanical milieu. The tension they exert on the ECM results in an extensive ECM remodeling potential, exemplified by the ability to realign extracellular fibronectin fibrils (104) and to contract collagenous ECM (36). VIC contraction increases ECM stiffness (105, 106). In addition, fibrotic ECM may provide less shielding from external mechanical forces that may be exerted on the VICs unabsorbed by the ECM.

## Molecular Mechanisms of Valvular Fibrosis and Potential to Reverse Remodeling

### Initiation of Myofibroblast Activation

The myofibroblast is the key cellular mediator of fibrosis and, when activated, serves as the primary collagen-producing cell type (58). Myofibroblasts are found all over the body, and their basic function and main characteristics are comparable (107–110). Initially, tissue damage takes place because of mechanical disruption or cell death in response to pathological conditions (i.e., hypoxia or overload). As a result, local inflammation leads to the secretion of cytokines, growth factors, and chemokines like connective tissue growth factor 2 (109), receptor activator of nuclear factor kappa B ligand (RANKL), tumor necrosis factor alpha (TNFα) (111), or connective tissue growth factor (CTGF) (112), resulting in the activation of local inactive cells of mesenchymal or fibroblastoid origin (108). The best-known and most potent master switch in myofibroblast differentiation is transforming growth factor beta 1 (TGFβ1). TGFβ1 induces phosphorylation and subsequent translocation of Smad2/3 into the nucleus where neo-expression of promyofibroblast genes is induced. The most prominent neo-expressed marker is α-SMA. Although α-SMA is not myofibroblast specific, its organization in stress fiber-like bundles *in vivo* is key to the myofibroblast-specific ability to contract the surrounding tissue (109). In addition to the expression of α-SMA, the expression of dozens of other proteins including cadherins, vimentin, fibronectin, collagens, MMP, and TIMPs is increased in myofibroblasts. In summary, mature myofibroblasts are characterized by altered adhesion ability, the production of profibrotic mediators, and active remodeling of the ECM surrounding them (113, 114). Importantly, TGFβ1 and the TGFβ1 receptor are actively expressed by mature myofibroblasts themselves. TGFβ1 is a physiological component of ECM; thus, it is released during myofibroblast-driven ECM remodeling (115). Consequently, myofibroblasts activation amplifies itself and can easily degenerate into a vicious cycle of TGFβ1-driven pathological ECM hyperproduction. This finally results in tissue thickening with dramatic consequences for the resident cell populations and the mechanical tissue characteristics as outlined before. Of note, targeting myofibroblastic activation may also reduce calcification (38) given that TGFβ1-driven VIC myofibroblastic differentiation may precede further differentiation to osteogenic phenotypes (116).

### Contribution of Other Signaling Pathways in Myofibroblast Activation and Regulation

Besides TGFβ1 signaling, other signaling pathways were found to be involved in VIC activation. Wnt/β-catenin signaling has an important function in embryogenesis of the heart. The reactivation of this signaling axis is thought to present a pathomechanism, which, in combination with TGFβ1, accelerates myofibroblasts differentiation. The interaction between these pathways directly correlates with increased extracellular stiffness (117). Extracellular stiffening also induces other signaling cascades, including RhoC, ROCK1/2, MEK/Erk1/2, and the translocation of myocardin-related transcription factor-A (MRTFa) into the nucleus to induce the expression of profibrotic CTGF (112).

VICs isolated from healthy aortic valves express toll-like receptors (TLR2 and 4), which were described to regulate the cellular inflammatory response, and the growth factor neurotrophin 3, which was shown to maintain the viability of existing neurons and to promote the growth and differentiation of new neurons (115). Stimulation of TLR4 with lipopolysaccharide activates the Akt and ERK1/2 pathway and increases the expression of neurotrophin 3. This activation subsequently results in VIC proliferation and increased collagen 3 and MMP9 production with implications for pathological ECM remodeling (118).

C-type natriuretic peptide (CNP), expressed by VECs and VICs, and the natriuretic peptide receptor 2 (NPR2) have also been implied in CAVD pathogenesis as mediators of myofibrogenesis and valvular fibrosis (119, 120). CNP increase was found to inhibit myofibroblast activation *in vitro* (120). In addition, in mice, it was shown that CNP signaling is mediated via NPR2 and that the loss of this receptor resulted in fibrosis, calcification, and aortic valve dysfunction (119).

Mutations in the receptor Notch1 were found to cause aortic valve calcification, leading the field's attention to this genetically highly preserved signaling pathway (121). While Notch1 is widely expressed throughout the body, mutation in humans and mice resulted in bicuspid aortic valves or severe CAVD (122). Notch1 was found to control several profibrotic pathways in response to altered shear stress (121). In zebrafish, Notch reactivation was induced by valvular damage and was crucial for the regeneration of the aortic valve (123).

### Neo-Angiogenesis in Heart Valve Fibrosis

Regulation of neo-angiogenesis accompanies heart valve fibrosis (124). While oxygen and nutrient requirement of resident cells in healthy valves is mainly covered by diffusion, supply in pathologically thickened tissue cannot sufficiently be maintained, resulting in cellular starvation, and hypoxia. Thus, the induction of blood vessel formation may be a feature of fibrotic remodeling. Elements of the renin–angiotensin signaling system (RAS) are modulators of angiogenesis and are expressed in the AV (125). The local homeostasis of the RAS is disturbed in CAVD (126). The gene expression of the angiotensin receptor 1 and of enzymes involved in angiotensin maturation and signaling (prorenin, renin, angiotensin converting enzyme, chymase, and cathepsin) are altered relative to control valves during late stages of CAVD (126). Importantly, apelin and the apelin receptor which are thought to promote signaling opposing the angiotensin pathway, were found to be upregulated during valve fibrosis (127). Angiotensin II signaling was further shown to crosstalk with TGFβ signaling involving canonical and non-canonical signaling pathways (107). The regulation of pro- and antifibrotic effects of elements of the RAS is complex and has been reviewed in detail previously (125).

### Epigenetics

Epigenetic mechanisms, namely, DNA methylation, posttranslational histone modification, chromatin remodeling, and differential expression of non-coding regulatory RNAs (microRNAs and long non-coding RNAs), are also involved in the initiation and progression of aortic valve fibrosis. This topic was recently comprehensively described in a review by Gošev et al. and will only be discussed very briefly and exemplarily here (128). DNA (cytosine-5-)-methyltransferase 3 beta (DNMT3B) activity was found significantly increased in dysfunctional aortic valves. It was accompanied by a general increase in global DNA methylation with more than 6000 altered methylation sites (129). Importantly, deactivation of DNMT3B slowed aortic stenosis progression (130). Histone deacetylases, which are involved in chromatin remodeling, with important implication for gene expression, were found to be regulated by shear stress. Thus, regulation of deacetylases may translate site-specific flow profile alterations in fibrotic heart valves into differential gene expression (131). Meanwhile, numerous non-coding regulatory RNAs were identified to be involved in the pathomechanisms in heart valve stenosis. More than 50 microRNAs that are up- or downregulated in diseased aortic valves were described (128, 132).

### Regression of Fibrosis, Molecular Targets, and Potential Pitfalls in Pharmaceutical Interventions

Fibrotic disease is reversible to a certain point as was described for liver cirrhosis (133) and pulmonary fibrosis (134, 135). The regression of fibrosis requires elimination of the initial triggering factors, degradation of excessive ECM, and inactivation of myofibroblasts (108). *In vivo*, myofibroblasts activity is counter-regulated by induction of apoptosis, senescence, dedifferentiation, and reprogramming (108, 110).

*In vitro*, myofibroblasts activation was reversed by polyunsaturated fatty acids (136), statins (137, 138), C-type natriuretic peptide (CNP) (120), fibroblast growth factor 2 (FGF2), and the FGF-receptor (139).

The recognition that TGFβ1 may mediate many of the profibrotic remodeling responses within the AV has led to a search for strategies to target this pathway. As TGFβ1 is a key cytokine involved in a myriad of signaling cascades throughout the body, direct targeting could result in deleterious off-target effects. However, downstream mediators may represent better targets. Exemplarily, TGFβ1 leads to an increase in the atypical cell adhesion protein cadherin-11 in VICs (113, 140), which was shown to mediate profibrotic responses in the lung (141), myocardium (142), and the liver (143). Cadherin-11 overexpression also induced extracellular matrix remodeling and calcification in murine AVs (144). Targeting cadherin-11 with a blocking antibody prevented AV remodeling in *Notch1*^+/−^ mutant mice (145).

Pharmacotherapy for CAVD has not yet translated into clinical practice. HMG-CoA inhibitors (statins) with their pleiotropic effects on cardiovascular biology failed to alleviate AS progression in major randomized controlled trials (RCTs). Nevertheless, these results should be carefully interpreted. First, it should be mentioned that these trials also included patients with bicuspid valves BAVs (146, 147). Second, the RCTs analyzed statin effects on an advanced disease stage. Given the fact that statins suppress myofibroblastic VICs *in vitro* (137, 138), one should assume that they can only impact on fibrosis. Fibrosis plays a major role in early-stage CAVD and in women with AS. Thus, statins may, however, be beneficial in the early treatment of patients at CAVD risk, who did not yet manifest severe disease (e.g., individuals with BAV or in women with AV sclerosis). Interestingly, the myofibroblast inhibitory effect of CNP may partially explain the statin effect, as statins were found to induce CNP expression in VICs (120).

Valvular fibrosis and neo-angiogenesis are connected on a functional and molecular basis as outlined above. Noteworthy, the crosstalk between angiotensin II signaling and TGFβ signaling can be inhibited using angiotensin receptor II blocking medication. These drugs are commonly used to treat hypertension, but *in vitro* Losartan also inhibited TGFβ signaling on SMAD-induced profibrotic gene expression (114).

Studies in cardiac hypertrophy have shown that the angiotensin II type 1 receptor colocalizes and requires the presence of a specific serotonergic receptor, 5-HT_2B_, for downstream signaling (148). Interestingly, an unintended agonism of 5-HT_2B_ resulted in severe valvulopathies in patients prescribed a specific class of ergot-derived dopamine agonists for Parkinson's disease and the diet drug combination Fenfluramine/Phentermine (149, 150). Studies showed that 5-HT_2B_ agonism leads to VIC proliferation and myofibroblast activation (151, 152). Consequently, these drugs were removed from the market, and protocols within the National Institute of Mental Health's Psychoactive Drug Screening Program to identify and prevent similar occurrences in the future were established. The recognition that 5-HT_2B_ can dramatically affect AV structure motivated studies to assess the therapeutic potential of targeting this receptor. Antagonism of 5-HT_2B_ was found to inhibit TGFβ1 signaling, including myofibroblast activation, through non-canonical interactions and inhibition of p38 MAPK (151). Accordingly, genetic ablation of 5-HT_2B_ improved AV function in *Notch1*^+/−^ mutant mice (153).

As mentioned before, TLRs and neutrophin 3 are involved in myofibroblast activation (118). Neutrophin neutralization using specific antibodies or inhibition of the downstream signaling was shown to inhibit the activation *in vitro*; whether a clinical application is thinkable needs to be evaluated. As Notch was found to be a key switch in valvular repair, the idea was proposed that artificially introduced changes in flow patterns on VECs in dysfunctional valves may activate Notch and the associated valve regeneration program (123).

For the sake of completeness, it needs to be mentioned that lifestyle modifications were also debated in the context of CAVD. Physical exercise was shown to attenuate early sclerotic lesion development in an experimental CAVD model *in vivo* (154). However, once lesions were established, exercise did not halt the progression of AV sclerosis (155). Non-smokers who only consumed low-to-moderate levels of alcohol were found to have a lower incidence of aortic stenosis in a 15.3-year follow-up study in 69,365 Swedish adults without cardiovascular disease at baseline (156). Ten years after smoking cessation, the risk to develop aortic stenosis was comparable to never smokers in this study. The molecular processes that underpin these observations remain widely elusive. However, the increase in cardio-protective high-density lipoprotein cholesterol, apolipoprotein A1, and adiponectin in light alcohol consumers (157) and the induction of TGFβ1 through nicotine (158) were discussed as causal mechanisms.

### Outlook and Implications for the Development of Pharmaceutical Intervention

Fibrotic disease in the liver and lungs was shown to be reversible (135, 159). However, the reversal of valvular fibrosis may be more complicated because even mild alterations in tissue composition have an immediate impact on tissue stiffness. The resulting elevated tissue stiffness will, in turn, provide higher resistance to blood flow, leading to altered, turbulent blood flow through the AV orifice. These fluid dynamics in turn may further drive pathological valve remodeling through mechanobiolological and inflammatory signaling. In addition, advanced fibrosis is often hypocellular, and it has been suggested that fibrosis becomes irreversible when the cellular mediators (the source of MMPs) are no longer present to reshape the ECM (160). Any intervention should thus occur early in the disease process when cell to ECM ratio is more favorable. In more progressed states, multiple targets may need to be addressed to restore ECM composition: ECM structure and layer microstructure concomitantly to reestablish physiological ECM homeostasis. Furthermore, proinflammatory pathways activated by high shear stress in severe AS may serve as valuable targets for pharmacological therapeutic interventions (161).

In summary, there could be a point of no return in the potential for reverse remodeling of the delicate layer architecture of the AV. However, inhibition of valvular fibrosis may still prove an attractive target, but a better understanding of fibrogenic AV biology is warranted. Major advances have been made defining the molecular contributors to the fibrotic CAVD stage using high-throughput technologies (48, 162, 163). These technologies and results will likely play a significant role in the discovery of novel molecular pathways with the potential to specifically target valvular fibrosis.

Importantly, there is mounting evidence that pathophysiology in women with CAVD is defined by a more fibrotic phenotype, while men are more prone to calcific AV disease. This finding has implications for the design of trials on pharmacological interventions in CAVD.

## Conclusions

Further dissection of the complex fibrotic aspect of CAVD pathobiology, considering the heterogeneous cellular environment, specialized layer structure, valvular biomechanics, and cellular mechanobiology, is needed to identify novel therapeutic targets. Definition of myofibroblastic VIC-specific markers or transcription factors may aid in the specific targeting of valvular myofibrogenesis. High-throughput technologies like scRNA-seq or single-nuclei RNA-seq will likely assist in the discovery of molecular targets and regulatory mechanisms that drive valvular fibrosis. Accounting for the sex-specific fibrocalcific CAVD pathogenesis, sex-specific therapeutic interventions may be warranted. Trials for therapeutic pharmacological interventions should be stratified according to underlying varying pathomechanisms. We expect that therapeutic intervention in fibrosis has an optimal window of opportunity that may be early in the course of the disease.

## Author Contributions

PB, LF, and FS performed the data search and drafted the manuscript. PL, HT, and JH critically revised the draft. All authors contributed to the conception of this work and approved the final version of the manuscript.

## Conflict of Interest

The authors declare that the research was conducted in the absence of any commercial or financial relationships that could be construed as a potential conflict of interest.
